# Plasma miRNAs in predicting radiosensitivity in non-small cell lung cancer

**DOI:** 10.1007/s13277-016-5052-8

**Published:** 2016-04-13

**Authors:** Xu Chen, Yanmei Xu, Xingyun Liao, Rongxia Liao, Luping Zhang, Kai Niu, Tao Li, Dezhi Li, Zhengtang Chen, Yuzhong Duan, Jianguo Sun

**Affiliations:** 1 Cancer Institute of PLA, Xinqiao Hospital, Third Military Medical University, Chongqing, 400037 China; 2 Oncology Department, Leshan People’s Hospital, Leshan, Sichuan 614000 China; 3 Medical English Department, College of Basic Medicine, Third Military Medical University, Chongqing, 400038 China

**Keywords:** NSCLC, miRNAs, Plasma, Radiosensitivity, Biomarker, Bioinformatics

## Abstract

**Background:**

Radioresistance of thoracic radiotherapy is a major bottleneck in the treatment of non-small cell lung cancer (NSCLC). Until now, there have been no effective biomarkers to predict the radiosensitivity.

**Purposes:**

Based on miRNA profile screened from NSCLC cell lines with different radiosensitivity, this study was conducted to explore the correlation between plasma miRNAs and radiotherapy response in NSCLC patients, and to identify biomarkers of the radiosensitivity in NSCLC.

**Methods:**

Differentially expressed genes were acquired from time-series gene expression profiles of radioresistant H1299 and radiosensitive H460 lung cancer cells (GSE20549). Potential miRNAs were screened from these differentially expressed genes by combining bioinformatics with GO analysis, pathway analysis, and miRNA prediction. A clinical observational study was performed to explore the correlation between candidate miRNAs and radiotherapy response. Stage IIIa–IV NSCLC patients who received two to four cycles of previous chemotherapy and underwent thoracic radiotherapy alone were included. Total RNA was purified from peripheral blood before radiotherapy, and plasma miRNAs were detected by real-time PCR (qRT-PCR). Then, tumor response, progression-free survival (PFS), and overall survival (OS) were acquired. Four miRNAs significantly different between effective and ineffective groups were further analyzed to obtain cutpoints from receiver operating characteristic (ROC) curves and the predictive value of radiosensitivity.

**Results:**

Candidate miRNAs included 14 miRNAs screened from radioresistant genes and five from radiosensitive genes. From Jan., 2013 to Dec., 2014, 54 eligible patients were enrolled with a median follow-up of 15.3 months (range 4.6 to 31.4) by the deadline of Aug. 31, 2015. Totally, there were no case of complete response (CR), 15 of partial response (PR), 35 of stable disease (SD), and 4 of progressive disease (PD). Eight patients had no progression and 19 patients were still alive. The median PFS and OS were 6.6 months (range 2.3 to 29.3) and 15.3 months (range 4.6 to 31.4), respectively. Four miRNAs (hsa-miR-98-5p, hsa-miR-302e, hsa-miR-495-3p, and hsa-miR-613) demonstrated a higher expression in effective group (CR + PR, 15 cases) than in ineffective group (SD + PD, 39 cases). Based on each cutpoint, objective response rate (ORR) was higher in miR-high group than in miR-low group. No miRNA showed correlation with median PFS or OS.

**Conclusion:**

Bioinformatical analysis and clinical verification reveal the correlation between plasma miRNAs and radiosensitivity in NSCLC patients. Plasma miRNAs represent novel biomarkers to predict radiotherapy response clinically.

**Electronic supplementary material:**

The online version of this article (doi:10.1007/s13277-016-5052-8) contains supplementary material, which is available to authorized users.

## Introduction

Lung cancer is the most common cancer worldwide with a 5-year survival rate less than 20 % [1, 2]. Non-small cell lung cancer (NSCLC) is the largest subgroup of lung cancer, approximately 70 % of which is unresectable stage III or IV disease. Thoracic radiotherapy has been the main treatment for advanced NSCLC patients [3]. However, radical thoracic radiotherapy usually cannot eradiate the tumor. Radioresistance is a major bottleneck in the treatment of tumor and its relapse [4]. The local recurrence rate in NSCLC is up to 60–70 % in 2 years after conventional fractionated radiotherapy (CFRT) (60 Gy/30 F/6 W) [5, 6]. Therefore, it is very critical to enhance the radiosensitivity of lung cancer so as to reduce the relapse.

The mechanism of radioresistance is very complicated, and it may involve pathological type, tumor microenvironment, radioresistant genes, DNA damage repair (DDR), tumor heterogenicity, individual differences, etc. [7, 8]. However, so far, no reliable biomarkers have been identified to stratify patients into different subgroups of radiotherapy response [9]. Recent studies indicate that miRNAs, a class of short non-coding RNAs, are involved in radioresistance [10–12]. As we know, miRNAs regulate cell cycle, cell proliferation, apoptosis, and tumor angiogenesis via targeting mRNA for degradation or inhibition of translation [13–16]. Moreover, miRNAs demonstrate a strong ability to regulate radiation response and DDR [10–12, 17]. All the above findings show that it is essential to obtain miRNAs associated with radiosensitivity. Thus, we attempted to explore the role of plasma miRNAs in predicting radiosensitivity in NSCLC.

In the current study, we screened miRNA profile associated with radiation response by comprehensive bioinformatical analysis of the time-series gene chips from irradiated NSCLC cell lines in the Gene Expression Omnibus (GEO) database. Then, we performed a clinical observational study to explore the correlation between plasma miRNAs and radiotherapy response in NSCLC patients. We also analyzed the predictive sensitivity and specificity of single miRNA or combined miRNAs. By doing this, we introduce a novel technology to predict radiosensitivity in NSCLC patients via detecting plasma miRNAs.

## Materials and methods

### Screening miRNA profile via bioinformatics

We searched the GEO database for gene chips of radiotherapy response in lung cancer and found GSE20549 (http://www.ncbi.nlm.nih.gov/geo/query/acc.cgi?acc=GSE20549) with the time-series gene expression profiles of radioresistant H1299 and radiosensitive H460 lung cancer cells in response to 2 Gy of ionizing radiation (IR). Comprehensive bioinformatics was then used to process the data, including GO analysis, pathway analysis, and miRNA prediction at home-made platform. Then, miRNA-mRNA networks were built to screen candidate miRNA profile associated with radiotherapy response [Supplementary material, Fig. 1a, b].

**Fig. 1:**
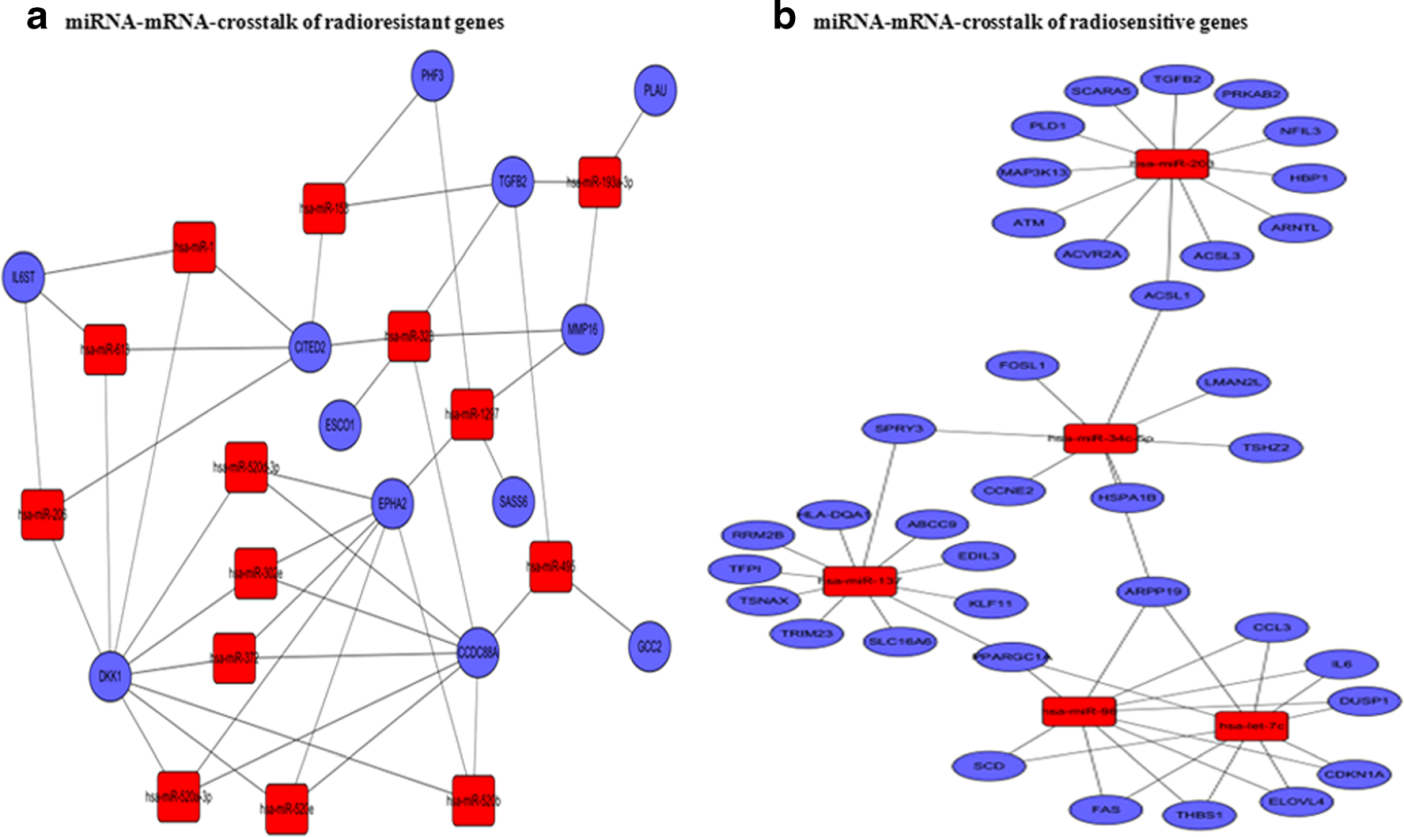
Bioinformatics of miRNA associated with radiosensitivity in NSCLC. **a** miRNA-gene-network (14 miRNAs associated with radioresistant genes). **b** miRNA-gene-network (5 miRNAs associated with radiosensitive genes). *Red rectangles* represent miRNAs, *blue circles* represent gene ontology, and *straight lines* represent the regulation between miRNAs and gene ontology

### Total RNA extraction from plasma

About 5 mL of non-coagulant blood was collected from every eligible patient. Plasma was isolated by centrifuging at 3000 rpm for 10 min at 4 °C, and stored at −80 °C. Total RNA extraction followed the manufacturer’s instruction of mirVana™ PARIS™ Kit (Ambion, USA). Briefly, chloroform was added to 200 μL of plasma, which was mixed with 2× denaturing solution and incubated on ice for 5 min. After centrifuging and removing the aqueous (upper) phase, 100 % ethanol was added, mixed thoroughly, pipetted onto the filter cartridge, and then centrifuged. After washing by 350 μL of wash solution three times, the filter was added with 100 μL of preheated (95 °C) nuclease-free water and centrifuged for 30 s to recover the RNA.

### Real-time RT-PCR (qRT-PCR) assay

We followed the previous protocol for primer design and qRT-PCR adopted by our institute [18]. Total RNA was reversely transcribed into cDNA with standard techniques (ABI, USA). Synthetic cel-39 small RNA (Ribobio, China) was used as an external control. The universal sense primer of miRNAs was 5′-GTGCAGGGTCCGAGGT-3′. Reverse transcription primer and antisense primer for qRT-PCR are listed in Supplementary Table S1. To enhance the efficacy of real-time PCR, pre-amplification of miRNAs was carried out using 2 × Taq PCR MasterMix (Qiagen, Germany) before real-time PCR. The pre-amplication was carried out as follows. Reaction system included 2 μL of cDNA, 1 μL of mixed antisense primers, 1 μL of universal sense primer, 12.5 μL of 2 × Taq PCR MasterMix, and 8.5 μL of nuclease-free water. Reaction condition was as follows: 94 °C for 3 min, 10 cycles of 94 °C for 30 s, 60 °C for 30 s, 72 °C for 1 min, and 72 °C for 15 min before being stored at −20 °C. Then, real-time PCR of cel-39 and miRNAs was performed using SYBR® Green PCR Master Mix (ABI, USA) as previously reported [18]. All the qRT-PCR reactions were repeated no less than three times.

### Patients

NSCLC patients confirmed by histological or cytological diagnosis were enrolled in the Oncology Department, Xinqiao Hospital, Third Military Medical University. TNM classification followed the International Classification of Diseases for Oncology [19]. Inclusion criteria included unresectable stage III or IV disease, two to four cycles of previous chemotherapy, unfitness for concurrent radiochemotherapy, Eastern Cooperative Oncology Group (ECOG) performance status (PS) 0–2, and an age from 18 to 75 years. Moreover, all eligible patients signed the informed consent form. Exclusion criteria included anti-tumor therapy within 4 weeks, previous thoracic radiotherapy and malfunction of vital organs. This study has been approved by the Ethics Committee of Third Military Medical University.

### Study design and workflow

**Fig. 2:**
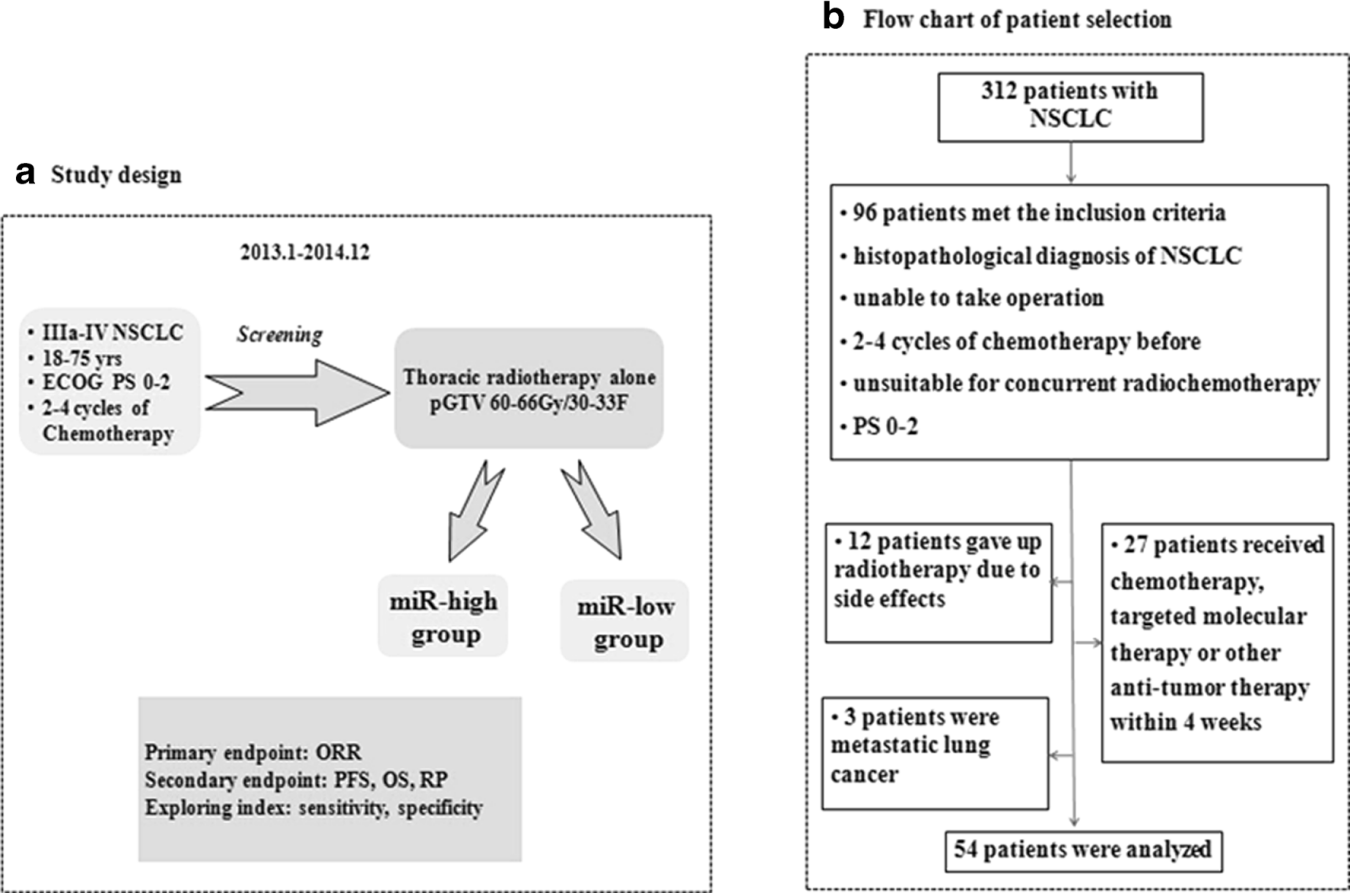
Study design and workflow. **a** Study design. **b** Flow chart of patient selection

The clinical observation study was performed from Jan., 2013 to Dec., 2014 (Fig. 2a). Every eligible patient received thoracic radiotherapy to the planning gross tumor volume (pGTV) (60–66 Gy/30–33 F/6–7 W). About 5 mL of non-coagulant blood was drawn before radiotherapy. Follow-up continued every month during and after radiotherapy by Aug. 31, 2015. Tumor response was estimated by RECIST 1.1. Median progression-free survival (PFS) and overall survival (OS) were recorded. The objective response rate (ORR) was defined as the proportion of patients with complete response (CR) or partial response (PR). PFS was calculated during the period from the first radiotherapy to disease progression. OS was calculated during the period from the first radiotherapy to death. Finally, the primary objective of ORR, secondary objectives of PFS, OS and radiation pneumonia (RP), and the exploring index of predictive sensitivity and specificity were analyzed.

### Statistical analysis

The expression of plasma miRNAs was compared by the independent-sample *t* test based on homogeneity test. The ORR comparison was analyzed by the standard chi-square test. The PFS and OS were estimated using the Kaplan-Meier method. Statistical analyses were performed with SPSS 17.0 (SPSS Inc., Chicago, IL, USA) at *P* < 0.05. The cutpoints of miRNAs were obtained from receiver operating characteristic (ROC) curves using MedCalc 15.10.

## Results

### Screening of candidate miRNA profile via bioinformatics

The GSE20549 discovered time-series gene expression profiles of radiotherapy reaction in NSCLC cell lines with 42 pieces of gene chips. A total of 12 samples were hybridized to Affymetrix Human Gene 1.0 Array gene chips. We screened differentially expressed genes associated with radioresistance and radiosensitivity by Two Class Dif. Based on the standards of gene significance level (*P* < 0.05) and false discovery rate (FDR < 0.05), 118 radioresistant genes and 1055 radiosensitive genes were primitively isolated (Supplementary differentially expressed genes.xls).

GO analysis was performed on all the above differentially expressed genes. After calculating the difference between gene significance level (*P* < 0.05) and FDR (FDR < 0.05), GO analysis exhibited 107 kinds of functions and 35 genes in radioresistant group, and 42 kinds of functions and 137 genes in radiosensitive group (Supplementary GO analysis.xls).

Pathway analysis was performed on all the above differentially expressed genes based on KEGG (Kyoto Encyclopedia of Genes and Genomes). Fisher’s exact test and chi-square test were used. After calculating the difference between gene significance level (*P* < 0.05) and FDR (FDR < 0.05), pathway analysis showed 5 kinds of functions and 10 genes in the radioresistant group and 18 kinds of functions and 143 genes in the radiosensitive group (Supplementary pathway analysis.xls).

All the above differentially expressed genes from both GO analysis and pathway analysis were analyzed using miRNA prediction tools. With the criterion of correlation index (*R* ≥ 0.5), we acquired 23 radioresistant genes and 26 potential miRNAs and 97 radiosensitive genes and 17 potential miRNAs, which are listed in Supplementary Table S2. By removing the intersecting miRNAs in both groups, two topology maps of miRNA-mRNA networks demonstrated the 14 miRNAs associated with radioresistant genes (Fig. 1a), and five miRNAs associated with radiosensitive genes (Fig. 1b), respectively. Specifically, the 14 miRNAs associated with radioresistant genes included hsa-miR-153-3p, hsa-miR-1-3p, hsa-miR-613, hsa-miR-372-3p, hsa-miR-302e, hsa-miR-495-3p, hsa-miR-206, hsa-miR-520a-3p, hsa-miR-328-3p, hsa-miR-520b, hsa-miR-1297, hsa-miR-520d-3p, hsa-miR-193a-3p and hsa-miR-520e, and the 5 miRNAs associated with radiosensitive genes included hsa-let-7c-5p, hsa-miR-98-5p, hsa-miR-203a-3p, hsa-miR-137, and hsa-miR-34c-5p.

### Patient characteristics

Collectively, 312 NSCLC patients were selected, among whom 96 met the inclusion criteria. However, 12 cases gave up radiotherapy due to severe side effects, and the other 27 cases were excluded because they received chemotherapy or targeted molecular therapy within the past 4 weeks; in addition, three cases had metastatic lung nodules. Eventually, the data of the remaining 54 patients are illustrated in our workflow (Fig. 2b).

All of the 54 patients (47 males and 7 females) with a median age of 60 years (range 31–75 years), 37 cases (68.5 %) were ever or current smokers. Seven patients were in stage IIIa (13.0 %), 22 (40.7 %) in stage IIIb, and 25 (46.3 %) in stage IV. It should be specified that the seven patients with stage IIIa disease did not undergo surgical resection because of the high risk of surgical intervention (Table 1).

**Table 1:** Patient characteristics

Characteristic	Total (*n* = 54)	Effective group (*n* = 15)	Ineffective group (*n* = 39)	*P* value
Age (years)				0.503
Median (range)	60 (31–75)	58.5 (31–71)	60.6 (38–75)	
Sex, *N* (%)				0.688
Male	47 (87.0)	14 (93.3)	33 (84.6)	
Female	7 (13.0)	1 (6.7)	6 (15.4)	
Histological type, *N* (%)				
SCC	32 (59.3)	9 (60.0)	23 (59.0)	0.945
Non-SCC	22 (40.7)	6 (40.0)	16 (41.0)	
Disease stage, *N* (%)				0.997
IIIa	7 (13.0)	2 (13.3)	5 (12.8)	
IIIb	22 (40.7)	6 (40.0)	16 (41.0)	
IV	25 (46.3)	7 (46.7)	18 (46.2)	
Smoking status, *N* (%)				0.884
Never	17 (31.5)	4 (26.7)	13 (33.3)	
Ever or current	37 (68.5)	11 (73.3)	26 (66.7)	
Radiation pneumonitis				0.811
Yes	31 (57.4)	9 (60.0)	22 (56.4)	
No	23 (42.6)	6 (40.0)	17 (43.6)	

### Tumor response and miRNA difference

**Fig. 3:**
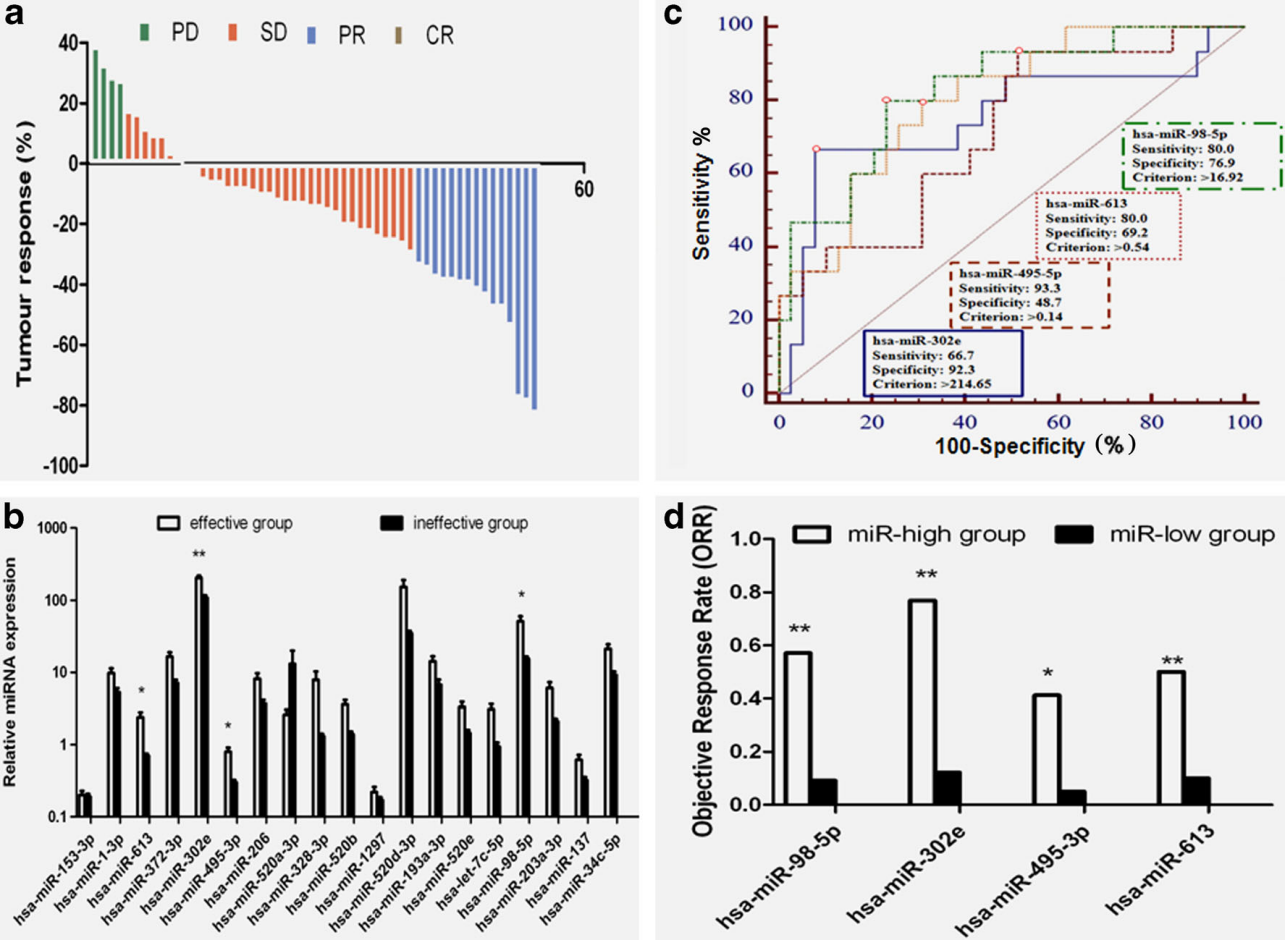
Tumor response and radiosensitivity prediction by plasma miRNAs. **a** Waterfall map of maximum tumor response in 54 patients. **b** Differentially expressed plasma miRNAs based on tumor response. Four miRNAs have a significant difference (*P < 0.05). **c** Cutpoints in receiver operating characteristic (ROC) curves of plasma miRNAs (*P < 0.05). **d** Tumor response based on cutpoints of plasma miRNAs (*P < 0.05, **P < 0.01)

Waterfall map showed maximum tumor response in 54 patients (Fig. 3a). Totally, 54 eligible patients reached 0 CR, 15 PR, 35 SD, and 4 PD. Eight patients had no progression and 19 patients were still alive. The median PFS and OS were 6.6 months (range 2.3 to 29.3) and 15.3 months (range 4.6 to 31.4), respectively. We divided all the eligible patients into effective group (CR + PR, 15 cases) and ineffective group (SD + PD, 39 cases). There was no baseline difference in patient characteristics between two groups in terms of age, sex, histological type, disease stage, and smoking status (*P* > 0.05, Table 1). Among these 19 candidate miRNAs, four miRNAs (hsa-miR-98-5p, hsa-miR-302e, hsa-miR-495-3p, and hsa-miR-613) demonstrated a higher expression in effective group than in ineffective group (*P* < 0.05) (Fig. 3b).

### Plasma miRNAs and ORR

Furthermore, ROC curves demonstrated the cutpoints of these four miRNAs as follows: hsa-miR-98-5p >0.14 (*P* = 0.0056), hsa-miR-302e >214.65 (*P* = 0.0035), hsa-miR-495-3p >16.92 (*P* < 0.0001), and hsa-miR-613 >0.54 (*P* < 0.0001) (Fig. 3c). We divided the 54 eligible patients into miR-high and miR-low groups based on the cutpoint criterion of each miRNA to compare the difference of ORR. We validated the significance of four above miRNAs in radiotherapy response (Fig. 3d). The ORRs of miR-high and miR-low groups were 63.6 vs. 18.6 % in hsa-miR-98-5p (*P* = 0.000), 76.9 vs. 12.2 % in hsa-miR-302e (*P* = 0.000), 41.2 vs. 5 % in hsa-miR-495-3p (*P* = 0.011), and 50.0 vs. 14.7 % in hsa-miR-613 (*P* = 0.003), respectively (Table 2).

**Table 2:** Correlation between Plasma miRNAs and tumor response

Tumor response	hsa-miR-98-5p		hsa-miR-302e		hsa-miR-495-3p		hsa-miR-613	
	miR-high *N* = 21, *n* (%)	miR-low *N* = 33, *n* (%)	miR-high *N* = 13, *n* (%)	miR-low *N* = 41, *n* (%)	miR-high *N* = 34, *n* (%)	miR-low *N* = 20, *n* (%)	miR-high *N* = 24, *n* (%)	miR-low *N* = 30, *n* (%)
CR	0 (0)	0 (0)	0 (0)	0 (0)	0 (0)	0 (0)	0 (0)	0 (0)
PR	12 (57.1)	3 (9.1)	10 (76.9)	5 (12.2)	14 (41.2)	1 (5.0)	12 (50.0)	3 (10.0)
SD	9 (42.9)	26 (78.8)	3 (23.1)	32 (78.0)	17 (50.0)	18 (90.0)	11 (45.8)	24 (80.0)
PD	0 (0)	4 (12.1)	0 (0)	4 (9.8)	3 (8.8)	1 (5.0)	1 (4.2)	3 (10.0)
ORR	12 (57.1)	3 (9.1)	10 (76.9)	5 (12.2)	14 (41.2)	1 (5.0)	12 (50.0)	3 (10.0)
*P* value	0.000		0.000		0.011		0.003	

### Plasma miRNAs and PFS, OS, RP

**Fig. 4:**
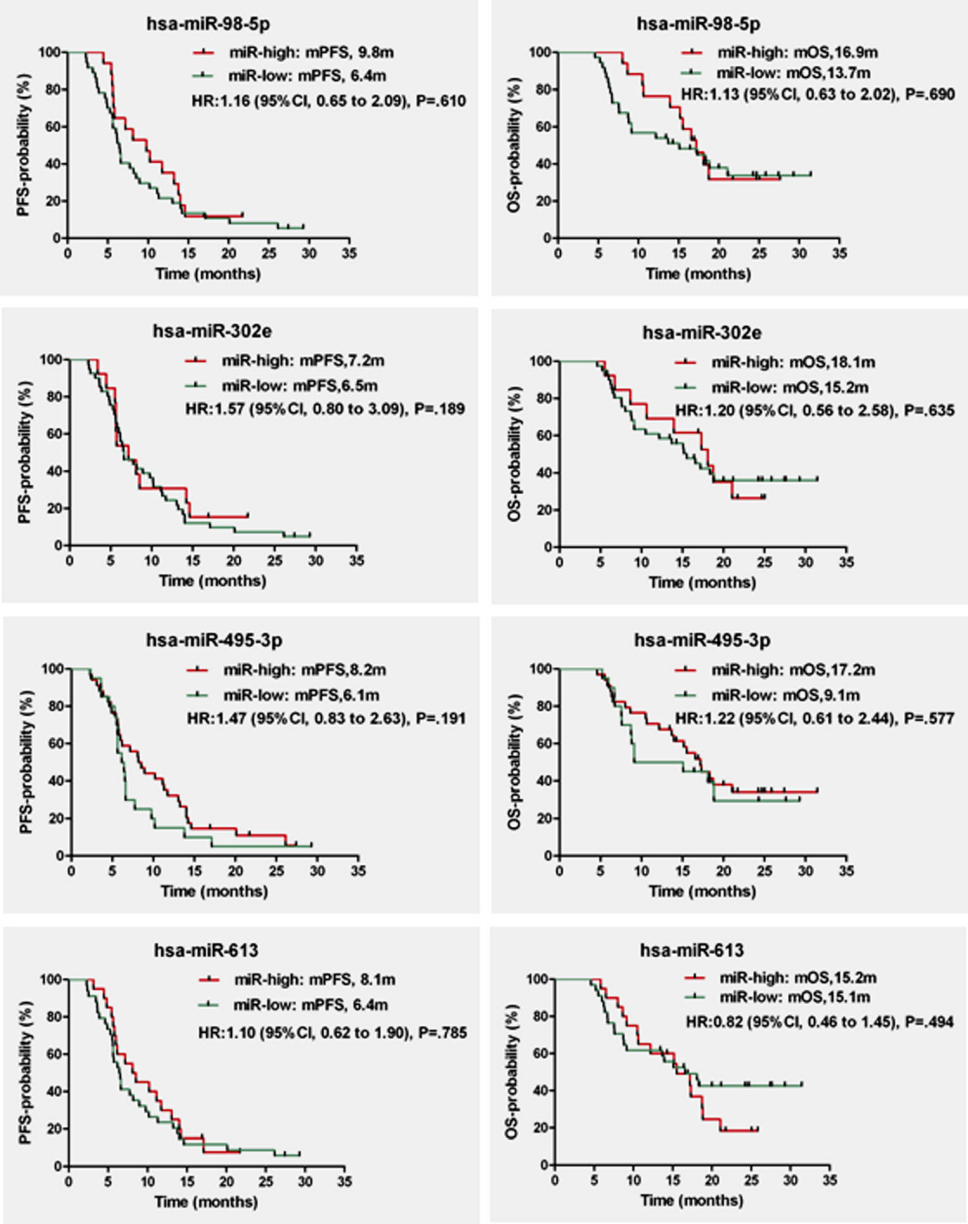
PFS and OS comparison based on miRNA level. Kaplan-Meier analysis of PFS and OS based on plasma miRNA level. No miRNA is associated with PFS or OS

The median follow-up was 15.3 months (range 4.6 to 31.4). Eight patients had no progression and nineteen patients were still alive. The median PFS and OS were 6.6 months (range 2.3 to 29.3) and 15.3 months (range 4.6 to 31.4), respectively. Comparison of PFS and OS difference in miR-high and miR-low groups of above miRNAs revealed that no miRNA was associated with PFS or OS (*P* > 0.05, Fig. 4).

RP of grades 1–4 occurred in 57.4 % of overall patients (31/54), in 60.0 % of patients in effective group (9/15), and in 56.4 % in ineffective group (22/35). There was no significant difference in RP between effective and ineffective groups (*P* > 0.05, Table 1). And there was no significant difference in RP between miR-high and miR-low groups, either, in hsa-miR-98-5p (57.1 vs. 36.4 %), hsa-miR-302e (53.8 vs. 41.5 %), hsa-miR-495-3p (44.1 vs. 45.0 %), and hsa-miR-613 (58.3 vs. 33.3 %), respectively (*P* > 0.05).

### Sensitivity and specificity of plasma miRNAs

Moreover, we calculated the predictive sensitivity and specificity of plasma miRNAs in radiotherapy response. The sensitivity and specificity ranged from 67 to 93 %, 49 to 90 %, respectively, in single miRNA; from 67 to 80 %, 72 to 97 %, respectively, in combination of two miRNAs; and from 60 to 67 %, 87 to 97 %, respectively, in combination of three miRNAs. And the sensitivity and specificity reached 60 and 97 % in combination of four miRNAs, respectively. With the addition of combination of miRNAs, the specificity increased while the sensitivity decreased. The conclusive results are listed in detail in Table 3.

**Table 3:** Sensitivity and specificity of plasma miRNA prediction

miRNAs	Prediction by single or combination of miRNAs														
hsa-miR-98-5p	+	−	−	−	+	−	−	+	+	−	+	+	−	+	+
hsa-miR-302e	−	+	−	−	−	+	−	+	−	+	+	−	+	+	+
hsa-miR-495-3p	−	−	+	−	+	+	+	−	−	−	+	+	+	−	+
hsa-miR-613	−	−	−	+	−	−	+	−	+	+	−	+	+	+	+
Sensitivity (%)	80	67	93	80	80	67	80	67	80	60	67	67	60	60	60
Specificity (%)	77	92	49	69	85	97	72	95	87	95	97	87	97	97	97

## Discussion

The morbidity and mortality of lung cancer remain high [20]. Radiotherapy is a key therapeutic strategy for lung cancer. However, most of patients suffer from the radioresistance which will decrease radiotherapy efficacy. Thus, it is urgent to overcome tumor radioresistance and enhance tumor radiosensitivity. By far, radiotherapy efficacy is mainly improved in two ways. On the one hand, high-end equipments and advanced technology are introduced, such as positron emission computed tomography (PET-CT), stereotactic radiotherapy (SBRT), and image-guide radiotherapy (IGRT). In spite of this, the local control of lung cancer is still not optimistic [21–23]. On the other hand, predictive biomarkers of radiosensitivity are studied as a useful strategy to monitor radiotherapy and optimize treatment plan in advance.

As known, miRNAs are a kind of small non-coding RNA molecules of about 22 nucleotides in length [24]. They are deemed to play a crucial role in the diagnosis, prognosis, and recurrence of lung cancer [25–27]. A number of studies have reported the potential clinical application of miRNAs in lung cancer radiotherapy. Some miRNAs are used to enhance anticancer therapy by regulating cell cycle, cell proliferation and apoptosis, DNA damage repair, tumor angiogenesis, and so on [28]. For example, miR-200c promoted radiosensitivity by downregulating oxidative response genes and inhibiting DNA repair in lung cancer [29]. Overexpression of miR-449a effectively increased irradiation-induced DNA damage and apoptosis, altered the cell cycle distribution and eventually led to sensitization of CL1-0 cell line to irradiation [30]. Upregulation of miR-138 enhanced radiosensitivity of lung cancer cells by inhibiting SENP1 and increasing apoptosis [31]. In addition, Let-7 and miR-25 have been proved to improve radiosensitivity in human tumors including lung cancer [9, 32]. Elevated evidence showed that circulating miRNAs could be potential biomarkers for early diagnosis and prognosis prediction in lung cancer [33, 34]. And miR-21 was verified as a biomarker for ionizing radiation in breast cancer [35]. However, there is no report about the role of plasma miRNAs in predicting radiosensitivity in NSCLC patients.

To our knowledge, the present research is the first one of its kind to screen miRNA profile and explore the correlation between plasma miRNAs and radiosensitivity in NSCLC patients. Four miRNAs (hsa-miR-98-5p, hsa-miR-302e, hsa-miR-495-3p, and hsa-miR-613) displayed the ability as indicators of radiosensitivity based on both tumor response criterion and miRNA level criterion. As we know, miRNAs usually downregulate target genes by inhibiting translation [12–15]. It is supposed that miRNAs associated with radioresistant genes may be radiosensitive candidates, and vice versa, miRNAs associated with radioresensitive genes may be radioresistant candidates. Actually, three miRNAs (hsa-miR-302e, hsa-miR-495-3p, and hsa-miR-613) associated with radioresistant genes demonstrated the predictive value. However, one miRNA (hsa-miR-98-5p) associated with radiosensitive genes also exhibited the function as an indicator of radiosensitivity. To our knowledge, miRNA expression usually changes during the process of disease progression and anti-tumor treatment. Possibly, radioresistant miRNAs would be downregulated, and radiosensitive miRNAs would be upregulated following the delivery of radiation in effective group. Nevertheless, it is not a good choice to predict the treatment effect by detecting the dynamic status of miRNA level during the delivery of radiotherapy. In current study, we aim to search for the biomarkers to predict the radiotherapy response before the treatment. The changes in miRNA after the radiotherapy are not our concern. And it is unnecessary to distinguish the function of a predicted miRNA before the radiotherapy. Thus, this study only reflects the relationship between plasma miRNAs and tumor response to radiotherapy. Future studies can be conducted to detect multiphase miRNA level during the radiotherapy and to explore the underlying mechanism to validate our results.

Consistent with our research, several previous studies showed that some above miRNAs were involved in the radio- or chemosensitivity. For example, miR-302 was inversely correlated with AKT1 and RAD52, two critical regulators of radioresistance. And miR-302a sensitized radioresistant breast cancer cells to radiotherapy in vitro and in vivo [36]. Overexpression of miR-495 increased the chemosensitivity of platinum-resistant lung adenocarcinoma cells by inhibiting copper-transporting P-type adenosine triphosphatase A (ATP7A), a gene of multi-drug resistance [37]. These findings confirm that the miRNAs mentioned above play an important role in radiotherapy reaction.

All eligible patients were strictly controlled to receive no chemotherapy, targeted molecular therapy or other anti-tumor therapy within 4 weeks before receiving radiotherapy. Our results show that the above four miRNAs are correlated with tumor response, but not PFS or OS, which could be caused by complex factors in PFS and OS, such as subsequent treatment and PS condition. According to Power and Precision v4, the power of PFS and OS ranges from 0.129 to 0.446 and 0.171 to 0.407, respectively, based on a one-sided significance level of 0.1. This low power may bring a false negative conclusion. Therefore, further prospective studies are needed, and a suitable sample size ought to be considered.

We also explored the predictive sensitivity and specificity of plasma miRNAs. Single miRNA displayed a relatively high sensitivity and a low specificity, and combined miRNAs showed a relatively low sensitivity and a high specificity. This result implies that plasma miRNAs can stratify NSCLC patients into radiosensitive and radioresistant subgroups. For the radioresistant subgroup, comprehensive intervention should be considered before radiotherapy, such as a higher dose or a concurrent strategy with other anticancer therapy [9].

## Conclusion

Our study provides the preliminary results that plasma miRNAs represent novel biomarkers to predict radiotherapy response in clinical settings. These findings lay the foundation for further validating the regulatory mechanism of target genes, GO and signal pathway in the radiosensitivity of NSCLC. Our study also provides guidance for clinical treatment. This study suggests that hsa-miR-98-5p, hsa-miR-302e, hsa-miR-495-3p, and hsa-miR-613 in plasma can be used as radiosensitivity indictors in NSCLC patients. Stage III multi-center clinical trial and further studies on the underlying mechanism are needed.

## Electronic supplementary material

Below is the link to the electronic supplementary material.

ESM 1 Supplementary Method (DOCX 33 kb)

ESM 2 Supplementary Tables (DOCX 35 kb)

ESM 3 Differential genes (XLS 196 kb)

ESM 4 GO analysis (XLSX 28 kb)

ESM 5 Pathway analysis (XLSX 17 kb)
