# Supplementary material for: Plasma miRNAs in predicting radiosensitivity in non-small cell lung cancer
Source: Tumour Biol. 2016 Apr 13;37(9):11927–36. doi: 10.1007/s13277-016-5052-8 (PMC5080326; doi:10.1007/s13277-016-5052-8)
Supplement: Supplementary file 1 — Supplementary Method (DOCX 33 kb) [file 13277_2016_5052_MOESM1_ESM.docx]

**Supplementary material**

**Data source**

We searched GEO database and found gene profiles of radiotherapy response in lung cancer (GSE20549, <http://www.ncbi.nlm.nih.gov/geo/query/acc.cgi?acc=GSE20549>). The time-series gene expression profiles of radioresistant H1299 and radiosensitive H460 lung cancer cells in response to 2 Gy of ionizing radiation (IR) were obtained.

**Two Class Dif**

The gene chip platform was Affymetrix Human Gene 1.0 ST Array. And there were 42 samples. We applied the RVM t-test to filter the differentially expressed genes in the control and the experimental groups, as it can effectively increase the degrees of freedom for small samples. After the significance analysis and FDR analysis, we selected the differentially expressed genes according to the P-value threshold (P < 0.05 and FDR < 0.05) [1-3].

**Gene Ontology (GO) analysis**

GO analysis was used to analyze the main function of the differentially expressed genes according to the Gene Ontology, the key functional classification of NCBI. [4,5]. Generally, Fisher’s exact test and $\chi^{2}$test were used to classify the GO category, and the false discovery rate (FDR) was calculated to correct the P value; the lower the FDR was, the smaller the error would be in judging the P value [6]. The FDR was calculated as$FDR=1-\frac{N_{k}}{T}$ ($N_{k}$ refers to the number of Fisher’s test, P values are less than$\chi^{2}$test P values). We computed P values for the GOs of all the differential genes. Enrichment provides a measure of the significance of the function: as the enrichment increases, the corresponding function is more specific, which helps us to find those GOs with more concrete function description in the experiment. Within the significant category, the enrichment Re was given by:$\mathrm{Re}=(n_{f}/n)/(N_{f}/N)$, where $n_{f}$ is the number of differential genes within the particular category, $n$is the total number of genes within the same category, $N_{f}$is the number of differential genes in the entire microarray, and $N$is the total number of genes in the microarray [7].

**Pathway Analysis**

Similarly, Pathway analysis was used to find out the significant pathway of the differential genes according to KEGG, Biocarta and Reatome. Still, we turned to the Fisher’s exact test and $\chi^{2}$test to select the significant pathway, and the threshold of significance was expressed as P value and FDR. The enrichment Re was calculated using the equation above [8-10].

**Gene-miRNA bioinformatics analysis**

Target genes were formed from both GO and Pathway analyses. The online software programs, miRBase (<http://www.mirbase.org/>), and home-made platform (Shanghai Genminix Informatics Co., Ltd., China.) were used for miRNA prediction of these target genes.

1. Wright GW, Simon RM. A random variance model for detection of differential gene expression in small microarray experiments. Bioinformatics. 2003; 19: 2448-55.

2. Yang H, Crawford N, Lukes L, Finney R, Lancaster M, Hunter KW. Metastasis predictive signature profiles pre-exist in normal tissues. Clin Exp Metastasis. 2005;22(7): 593-603.

3. Clarke R, Ressom HW, Wang A, Xuan J, Liu MC, Gehan EA, et al. The properties of high-dimensional data spaces: implications for exploring gene and protein expression data. Nat Rev Cancer. 2008; 8(1): 37-49.

4. The Gene Ontology (GO) project in 2006. Nucleic Acids Res, 2006; 34: D322-26.

5. Ashburner M, Ball CA, Blake JA, Botstein D, Botstein D, Butler H, Cherry JM, et al. Gene ontology: tool for the unification of biology. The Gene Ontology Consortium Nature genetics. 2000; 25(1): 25-29.

6. Dupuy D, Bertin N, Hidalgo CA, Venkatesan K, Tu D, Lee D, et al. Genome-scale analysis of in vivo spatiotemporal promoter activity in Caenorhabditis elegans. Nat Biotechnol. 2007; 25(6): 663-68.

7. Schlitt T, Palin K, Rung J, Dietmann S, Lappe M, Ukkonen E. et al. From gene networks to gene function. Genome Res. 2003; 13(12): 2568-76.

8. Kanehisa M, Goto S, Kawashima S, Okuno Y, Hattori M. The KEGG resource for deciphering the genome. Nucleic Acids Res. 2004; 32(suppl 1): D277-80.

9. Yi M, Horton JD, Cohen JC, Hobbs HH, Stephens RM. Whole Pathway Scope: a comprehensive pathway-based analysis tool for high-throughput data. BMC Bioinformatics. 2006; 7(1): 1.

10. Draghici S, Khatri P, Tarca AL, Amin K, Done A, Voichita C, et al... A systems biology approach for pathway level analysis. Genome Res. 2007;17(10): 1537-45.
