# Supplementary material for: Plasma miRNAs in predicting radiosensitivity in non-small cell lung cancer
Source: Tumour Biol. 2016 Apr 13;37(9):11927–36. doi: 10.1007/s13277-016-5052-8 (PMC5080326; doi:10.1007/s13277-016-5052-8)
Supplement: Supplementary file 2 — Supplementary Tables (DOCX 35 kb) [file 13277_2016_5052_MOESM2_ESM.docx]

**Table S1 RT and antisense primers of miRNAs for qRT-PCR**

| **miRNAs** | **RT primers** | **antisense primers** |
| --- | --- | --- |
| hsa-miR-153-3p | 5′-gtcgtatccagtgcagggtccgaggtattcgcactggatacgacgatcac-3′ | 5′-ttacgttgcatagtcacaaaagtga-3′ |
| hsa-miR-1-3p | 5′-gtcgtatccagtgcagggtccgaggtattcgcactggatacgacatacat-3′ | 5′-gctgctggaatgtaaagaagtatgtat-3′ |
| hsa-miR-613 | 5′-gtcgtatccagtgcagggtccgaggtattcgcactggatacgacggcaaa-3′ | 5′-cactgtgctaggaatgttccttct-3′ |
| hsa-miR-372-3p | 5′-gtcgtatccagtgcagggtccgaggtattcgcactggatacgacacgctc-3′ | 5′-ctgaaagtgctgcgacatttg-3′ |
| hsa-miR-302e | 5′-gtcgtatccagtgcagggtccgaggtattcgcactggatacgacaagcat-3′ | 5′-atactcgtaagtgcttccatgctt-3′ |
| hsa-miR-495-3p | 5′-gtcgtatccagtgcagggtccgaggtattcgcactggatacgacaagaag-3′ | 5′-aacgtaaacaaacatggtgcact-3′ |
| hsa-miR-206 | 5′-gtcgtatccagtgcagggtccgaggtattcgcactggatacgacccacac-3′ | 5′-ctatggaatgtaaggaagtgtgtgg-3′ |
| hsa-miR-520a-3p | 5′-gtcgtatccagtgcagggtccgaggtattcgcactggatacgacacagtc-3′ | 5′-aaagtgcttccctttggactgt-3′ |
| hsa-miR-328-3p | 5′-gtcgtatccagtgcagggtccgaggtattcgcactggatacgacacggaa-3′ | 5′-tctggccctctctgccct-3′ |
| hsa-miR-520b | 5′-gtcgtatccagtgcagggtccgaggtattcgcactggatacgacccctct-3′ | 5′-agtgcaaagtgcttccttttagag-3′ |
| hsa-miR-1297 | 5′-gtcgtatccagtgcagggtccgaggtattcgcactggatacgaccacctg-3′ | 5′-ctgcttcgttcaagtaattcaggt-3′ |
| hsa-miR-520d-3p | 5′-gtcgtatccagtgcagggtccgaggtattcgcactggatacgacacccac-3′ | 5′-aaagtgcttctctttggtgggt-3′ |
| hsa-miR-193a-3p | 5′-gtcgtatccagtgcagggtccgaggtattcgcactggatacgacactggg-3′ | 5′-aactggcctacaaagtcccagt-3′ |
| hsa-miR-520e | 5′-gtcgtatccagtgcagggtccgaggtattcgcactggatacgaccctca-3′ | 5′-ctaaagtgcttcctttttgaggg-3′ |
| hsa-let-7c-5p | 5′-gtcgtatccagtgcagggtccgaggtattcgcactggatacgacaaccat-3′ | 5′-cgctgaggtagtaggttgtatggt-3′ |
| hsa-miR-98-5p | 5′-gtcgtatccagtgcagggtccgaggtattcgcactggatacgacaacaat-3′ | 5′-atccgttgaggtagtaagttgtattgt-3′ |
| hsa-miR-203a-3p | 5′-gtcgtatccagtgcagggtccgaggtattcgcactggatacgacctagtg-3′ | 5′-gtcgtgaaatgtttaggaccactag-3′ |
| hsa-miR-137 | 5′-gtcgtatccagtgcagggtccgaggtattcgcactggatacgacctacgc-3′ | 5′-tcttcgttattgcttaagaatacgc-3′ |
| hsa-miR-34c-5p | 5′-gtcgtatccagtgcagggtccgaggtattcgcactggatacgacgcaatc-3′ | 5′-tattcaggcagtgtagttagctgatt-3′ |
| cel-miR-39-3p | 5′-gtcgtatccagtgcagggtccgaggtattcgcactggatacgaccaagct-3′ | 5′-tcaccgggtgtaaatcagctt-3′ |

**Table S2 Candidate miRNAs and radiation associated genes**

| **Radioresistant genes** | | | | |  | **Radiosensitive genes** | | | | |
| --- | --- | --- | --- | --- | --- | --- | --- | --- | --- | --- |
| **miRNA** | **Degree** | **Correlation**  **Index (R)** | **Gene** | **Degree** |  | **miRNA** | **Degree** | **Correlation**  **Index (R)** | **Gene** | **Degree** |
| hsa-miR-181d | 6 | 1 | CCDC88A | 15 |  | hsa-miR-590-3p | 16 | 1 | TP53INP1 | 19 |
| hsa-miR-328-3p | 5 | 0.8333333 | DKK1 | 13 |  | hsa-miR-181d | 15 | 0.9375 | PPARGC1A | 16 |
| hsa-miR-128 | 4 | 0.6666667 | CITED2 | 11 |  | hsa-miR-429 | 15 | 0.9375 | DNAJC3 | 15 |
| hsa-miR-1297 | 4 | 0.6666667 | TGFB2 | 11 |  | hsa-miR-301b | 13 | 0.8125 | DKK1 | 13 |
| hsa-miR-301b | 4 | 0.6666667 | MMP16 | 10 |  | hsa-miR-203a-3p | 12 | 0.75 | ACVR2A | 12 |
| hsa-miR-1-3p | 3 | 0.5 | EPHA2 | 8 |  | hsa-miR-519d | 12 | 0.75 | ARPP19 | 12 |
| hsa-miR-152 | 3 | 0.5 | PLK2 | 7 |  | hsa-miR-137 | 11 | 0.6875 | C1GALT1 | 12 |
| hsa-miR-153-3p | 3 | 0.5 | GCC2 | 6 |  | hsa-let-7c-5p | 10 | 0.625 | NCOA7 | 12 |
| hsa-miR-193a-3p | 3 | 0.5 | PHF3 | 6 |  | hsa-miR-98-5p | 10 | 0.625 | TGFB2 | 11 |
| hsa-miR-206 | 3 | 0.5 | AHCTF1 | 5 |  | hsa-miR-320a | 9 | 0.5625 | SPRY3 | 10 |
| hsa-miR-302e | 3 | 0.5 | ATL3 | 5 |  | hsa-miR-320b | 9 | 0.5625 | BCL6 | 9 |
| hsa-miR-320a | 3 | 0.5 | IL6ST | 5 |  | hsa-miR-320c | 9 | 0.5625 | ACSL1 | 8 |
| hsa-miR-320b | 3 | 0.5 | LIF | 4 |  | hsa-miR-320d | 9 | 0.5625 | IL8 | 8 |
| hsa-miR-320c | 3 | 0.5 | ESCO1 | 3 |  | hsa-miR-128 | 8 | 0.5 | LEFTY2 | 8 |
| hsa-miR-320d | 3 | 0.5 | RFC1 | 3 |  | hsa-miR-152 | 8 | 0.5 | SLC16A6 | 8 |
| hsa-miR-372-3p | 3 | 0.5 | SASS6 | 3 |  | hsa-miR-34c-5p | 8 | 0.5 | SLC40A1 | 8 |
| hsa-miR-429 | 3 | 0.5 | CYR61 | 2 |  | hsa-miR-520c-3p | 8 | 0.5 | ACSL3 | 7 |
| hsa-miR-495-3p | 3 | 0.5 | HSPA1B | 2 |  |  |  |  | CDKN1A | 7 |
| hsa-miR-519d | 3 | 0.5 | ID2 | 2 |  |  |  |  | PER2 | 7 |
| hsa-miR-520a-3p | 3 | 0.5 | PLAU | 2 |  |  |  |  | SCD | 7 |
| hsa-miR-520b | 3 | 0.5 | SKIL | 2 |  |  |  |  | SOS2 | 7 |
| hsa-miR-520c-3p | 3 | 0.5 | SPRY4 | 2 |  |  |  |  | PRKAB2 | 6 |
| hsa-miR-520d-3p | 3 | 0.5 | DPY19L2 | 1 |  |  |  |  | RAB39B | 6 |
| hsa-miR-520e | 3 | 0.5 |  |  |  |  |  |  | ST3GAL1 | 6 |
| hsa-miR-590-3p | 3 | 0.5 |  |  |  |  |  |  | THBS1 | 6 |
| hsa-miR-613 | 3 | 0.5 |  |  |  |  |  |  | ELOVL4 | 5 |
|  |  |  |  |  |  |  |  |  | GALNT4 | 5 |
|  |  |  |  |  |  |  |  |  | HBP1 | 5 |
|  |  |  |  |  |  |  |  |  | ITGAV | 5 |
|  |  |  |  |  |  |  |  |  | KLF11 | 5 |
|  |  |  |  |  |  |  |  |  | MOSPD1 | 5 |
|  |  |  |  |  |  |  |  |  | PIGK | 5 |
|  |  |  |  |  |  |  |  |  | PIK3C2A | 5 |
|  |  |  |  |  |  |  |  |  | RHOBTB3 | 5 |
|  |  |  |  |  |  |  |  |  | JUN | 4 |
|  |  |  |  |  |  |  |  |  | CAMK2D | 4 |
|  |  |  |  |  |  |  |  |  | MAP3K13 | 4 |
|  |  |  |  |  |  |  |  |  | NKX2-4 | 4 |
|  |  |  |  |  |  |  |  |  | RRM2B | 4 |
|  |  |  |  |  |  |  |  |  | SESN1 | 4 |
|  |  |  |  |  |  |  |  |  | ABCC9 | 3 |
|  |  |  |  |  |  |  |  |  | ARNTL | 3 |
|  |  |  |  |  |  |  |  |  | CCNE2 | 3 |
|  |  |  |  |  |  |  |  |  | DUSP1 | 3 |
|  |  |  |  |  |  |  |  |  | EDIL3 | 3 |
|  |  |  |  |  |  |  |  |  | FCGR3A | 3 |
|  |  |  |  |  |  |  |  |  | FOSL1 | 3 |
|  |  |  |  |  |  |  |  |  | NRBF2 | 3 |
|  |  |  |  |  |  |  |  |  | PGAP1 | 3 |
|  |  |  |  |  |  |  |  |  | PLD1 | 3 |
|  |  |  |  |  |  |  |  |  | RPS6KA6 | 3 |
|  |  |  |  |  |  |  |  |  | SPOCK1 | 3 |
|  |  |  |  |  |  |  |  |  | TNFRSF11B | 3 |
|  |  |  |  |  |  |  |  |  | ARMCX3 | 2 |
|  |  |  |  |  |  |  |  |  | ATM | 2 |
|  |  |  |  |  |  |  |  |  | ATP6V1G2 | 2 |
|  |  |  |  |  |  |  |  |  | CCL3 | 2 |
|  |  |  |  |  |  |  |  |  | ERRFI1 | 2 |
|  |  |  |  |  |  |  |  |  | FADS2 | 2 |
|  |  |  |  |  |  |  |  |  | FAS | 2 |
|  |  |  |  |  |  |  |  |  | HLA-DQA1 | 2 |
|  |  |  |  |  |  |  |  |  | HSPA1B | 2 |
|  |  |  |  |  |  |  |  |  | IL1A | 2 |
|  |  |  |  |  |  |  |  |  | IL1R1 | 2 |
|  |  |  |  |  |  |  |  |  | IL6 | 2 |
|  |  |  |  |  |  |  |  |  | KIF20B | 2 |
|  |  |  |  |  |  |  |  |  | LMAN2L | 2 |
|  |  |  |  |  |  |  |  |  | NUDT12 | 2 |
|  |  |  |  |  |  |  |  |  | PRKAA2 | 2 |
|  |  |  |  |  |  |  |  |  | SHC4 | 2 |
|  |  |  |  |  |  |  |  |  | TFPI | 2 |
|  |  |  |  |  |  |  |  |  | TOM1L1 | 2 |
|  |  |  |  |  |  |  |  |  | TRIM23 | 2 |
|  |  |  |  |  |  |  |  |  | TSHZ2 | 2 |
|  |  |  |  |  |  |  |  |  | TSNAX | 2 |
|  |  |  |  |  |  |  |  |  | CACNA2D1 | 1 |
|  |  |  |  |  |  |  |  |  | CBLB | 1 |
|  |  |  |  |  |  |  |  |  | CCL20 | 1 |
|  |  |  |  |  |  |  |  |  | CCPG1 | 1 |
|  |  |  |  |  |  |  |  |  | CD226 | 1 |
|  |  |  |  |  |  |  |  |  | CD24 | 1 |
|  |  |  |  |  |  |  |  |  | DNAJB4 | 1 |
|  |  |  |  |  |  |  |  |  | DPY19L2 | 1 |
|  |  |  |  |  |  |  |  |  | HIST1H4D | 1 |
|  |  |  |  |  |  |  |  |  | HSPA6 | 1 |
|  |  |  |  |  |  |  |  |  | ID1 | 1 |
|  |  |  |  |  |  |  |  |  | IL18 | 1 |
|  |  |  |  |  |  |  |  |  | MBTPS1 | 1 |
|  |  |  |  |  |  |  |  |  | NEU1 | 1 |
|  |  |  |  |  |  |  |  |  | NFIL3 | 1 |
|  |  |  |  |  |  |  |  |  | PAPPA2 | 1 |
|  |  |  |  |  |  |  |  |  | RIPK2 | 1 |
|  |  |  |  |  |  |  |  |  | SCARA5 | 1 |
|  |  |  |  |  |  |  |  |  | SENP7 | 1 |
|  |  |  |  |  |  |  |  |  | SERPINB2 | 1 |
|  |  |  |  |  |  |  |  |  | SERPINB9 | 1 |
|  |  |  |  |  |  |  |  |  | SLC25A20 | 1 |
